# Construction and Evaluation of High-Efficiency Tannase-Producing Strains

**DOI:** 10.3390/microorganisms14061233

**Published:** 2026-05-30

**Authors:** Yuan Gao, Chenguang Hu, Wurilege Wei, Delhei Urjid, Yuchao Hu, Xiaojuan Zhao, Yang Liu, Guoqing Guo, Surigalatu Wang, Feng Tian, Jianyong Liang, Jiuyue Li, Hai Jin, Shuyuan Xue

**Affiliations:** 1 Inner Mongolia Academy of Agricultural and Animal Husbandry Sciences, Hohhot 010031, China; may5june6@yeah.net (Y.G.); baron_hcg@sina.com (C.H.); weiwurilege@163.com (W.W.); delhi98@163.com (D.U.); yuchaohu1994@163.com (Y.H.); m15648192392@163.com (X.Z.); 15661271930@163.com (Y.L.); guoqingguo2024@163.com (G.G.); surigalatu2025@163.com (S.W.); tianfeng1309802@126.com (F.T.); liangjy0403@163.com (J.L.); lijy009@163.com (J.L.); jinhaicnm@vip.sina.com (H.J.); 2 Inner Mongolia Agricultural University, Hohhot 010018, China

**Keywords:** tannase, *Bacillus subtilis*, recombinant strains, pHT43, p43NMK

## Abstract

The low production efficiency of tannase and the insufficient utilization of high-tannin feed resources form the research background and research significance of this study. In this experiment, the tannase sequence TanLpl from *Lactiplantibacillus plantarum* ATCC14917T (obtained from a microbial culture collection) was selected. These sequences were respectively integrated into the expression systems of *Bacillus subtilis* 168 (BS168) and *Bacillus subtilis* WB600 (WB600) through plasmids TanLpl-p43NMK and TanLpl-pHT43. This successfully constructed three tannase-producing strains: TanLpl-p43NMK-*Bacillus subtilis* 168 (BS168(p43NMK)), TanLpl-pHT43-*Bacillus subtilis* 168 (BS168(pHT43)), and TanLpl-pHT43-*Bacillus subtilis* WB600 (WB600(pHT43)). An evaluation of the recombinant strains’ growth characteristics, expression stability, and enzymatic properties revealed that all three strains reached the stationary phase after 18 h of growth, with no significant differences in growth rate compared to the parental strains. At the 10th generation of subculture, the plasmid loss rate of BS168(p43NMK) was significantly higher than that of BS168(pHT43) or WB600(pHT43) (*p* < 0.05). The optimal temperature for tannase activity in all three recombinant strains was 30 °C, with an optimal pH value of 5.0. Under these conditions, the tannase activities were 68.81 U/mL, 397.36 U/mL, and 461.12 U/mL, respectively. The recombinant strain WB600(pHT43) exhibited superior expression stability and enzyme production capability compared to the other two strains. The research on the heterologous expression of tannase and its application in feed utilization has important theoretical and practical significance: it enriches the technical system for the heterologous expression of functional enzymes in *Bacillus subtilis*, provides new ideas for the efficient production of industrial enzymes, and promotes the development of bio-manufacturing technology.

## 1. Introduction

Tannins are naturally occurring plant compounds that are extensively present in feeding patterns and possess varying biological activities. Excess tannins can inhibit nutrient digestion and absorption, damage the intestinal mucosal barrier, suppress immune function, slow growth rate and even reduce the reproductive performance of animals. Therefore, various approaches have been used to lower and/or remove tannins in high-tannin-containing feeds, especially in areas where feed sources are limited and animals rely on feed with high tannin content [1]. The application of tannase is one of them [2].

Tannase (tannin acylhydrolase) is an enzyme produced by tannase-producing microorganisms, primarily fungi and bacteria. Fungal-derived tannases generally exhibit higher enzymatic activity compared to their bacterial counterparts [3,4]. However, the production efficiency of native tannase-producing fungi and bacteria is insufficient to support the industrial application of this enzyme. Therefore, there is a need to develop an efficient heterologous expression of tannase to enhance enzyme production such that could facilitate the utilization of high-tannin-containing feed [5]. Jingya Wu et al. [6] cloned a putative gene encoding the subtype B tannase (Gt-Tan) from *Galactobacillus timonensis* and expressed heterologously in *Escherichia coli* BL21 (DE3) cells. The Gt-Tan was purified using metal-affinity chromatography and exhibited a monomeric structure with a molecular weight of 55 kDa. Gt-Tan showed optimal activity at a temperature of 50°C and a pH value of 6.0. Nalapat Leangnim et al. [7] isolated nine tannase-producing yeasts; all tannases were produced within the same production yield (11 mU/mL). Rodríguez et al. [8] isolated *Lactiplantibacillus plantarum* CECT 784T (also known as ATCC14917T), whose cell-free extract demonstrated maximum tannase activity at pH 5.0 and 30 °C. The tannase gene sequence *TanLpl* from *L. plantarum* ATCC14917T contains an open reading frame of 1410 bp, encoding a 469-amino acid protein. Purified tannase is a monomeric polypeptide with a molecular weight of approximately 50 kDa. The enzymatic activity of TanLpl-encoded tannase surpasses that of other bacterial tannases, displaying a specific activity of 84.34 U/mg at pH 5.0 and 30 °C after chromatographic purification, with a maximum specific activity of 131 U/mg (equivalent to 214 U/mL) at pH 5.0 and 30 °C. Pulido et al. [9] demonstrated that *L. plantarum* harboring *TanLpl* can release proteins and metal ions chelated by tannins in fermentation substrates, utilizing these liberated nutrients. This confers ecological advantages over non-tannase-producing bacteria and tannase-producing fungi during the early stages of feed fermentation. Consequently, the *TanLpl* gene from *L. plantarum* ATCC14917T is ideally suited for caragana-fermented feed production [10].

*Bacillus subtilis* has been an exceptional expression host due to its beneficial properties and utility in heterologous protein production [11]. Some advantages of using *B. subtilis* as an expression host include short growth cycle, non-pathogenicity [12], the absence of exotoxin and endotoxin production, broad cultivation adaptability, robust extracellular protein secretion capacity, etc. [13]. These attributes make *B. subtilis* an optimal host for industrial-scale heterologous protein expression. Shuhei Ueda et al. employed *Bacillus subtilis* Rik1285 as the expression host to heterologously express the tannase genes *tanLpl*, *tanLpa*, and *tanLpe* derived from *Lactiplantibacillus plantarum*, *L. paraplantarum*, and *L. pentosus*, respectively. Their study was restricted to the characterization of enzymatic properties, without further optimization for high-level enzyme activity expression [14].

The tannase gene sequence *TanLpl* from *Lactiplantibacillus plantarum* ATCC14917T (GenBank accession number: AB379685) was added into the expression systems of *Bacillus subtilis* 168 and *Bacillus subtilis* WB600. This was achieved using an integrated plasmid method, aiming to obtain recombinant strains capable of high-efficiency tannase production. This study provides a foundation for utilizing caragana as feed material.

## 2. Materials and Methods

### 2.1. Materials

The strains, plasmids, markers, kits and their sources used in this experiment are listed in Table 1.

The culture media, electroporation buffer and their compositions are listed in Table 2.

### 2.2. Primer Design and Synthesis

Primers used in this study (Table 3) were designed based on the TanAn and TanLpl (GenBank accession no. AB379685) gene sequences in GenBank. The primers and TanLpl gene sequence were synthesized by Sangon Biotech (Shanghai, China) Co., Ltd.

### 2.3. Recombinant Plasmid Construction

#### 2.3.1. Construction of Plasmid TanLpl-p43NMK

The *TanLpl* sequence was amplified in PCR with TanLpl-F and TanLpl-R primers using *Lactiplantibacillus plantarum* ATCC14917T genomic DNA, prepared as above, as template. The amplified product was then electrophoresed on agarose gel, following the manufacturer’s protocol, and the target band was extracted and purified twice, following the manufacturer’s protocol. This was followed by fusing TanLpl-p43-F/TanLpl-p43-R. PCR was performed using the *TanLpl* amplicon as the template, with primers containing the P43 promoter, and the target band was recovered and purified for downstream use. The p43NMK vector backbone was individually amplified from the p43NMK plasmid using primers p43NMK-HindIIIF and p43-R, which was followed by gel electrophoresis and the purification of the target band.

The PCR reactions were performed using a Bio-Rad T100 Thermal Cycler (Bio-Rad Laboratories, Hercules, CA, USA). The reaction components and dosages are shown in Table 4. The reaction parameters were determined based on previous studies. The amplification procedures were set as follows: initial denaturation at 94 °C for 5 min, followed by 30 cycles of denaturation at 94 °C for 30 s, annealing at 55 °C for 30 s, and extension at 68 °C for 30 s. A final extension was carried out at 72 °C for 5 min, and the reaction mixtures were maintained at 4 °C until subsequent treatments.

The purified *TanLpl* amplicon and the p43NMK vector backbone PCR products were ligated using a recombinant cloning kit of Easy Geno Assembly Mix (Table 1), in accordance with the manufacturer’s protocol. The reaction mixtures are described in Table 5.

The recombinant plasmids were then transformed into *Escherichia coli* DH5α competent cells. The preparation of competent cells and transformation operations for *Bacillus subtilis* were conducted following standard protocols [15]. The transformed strains were subsequently spread onto LB agar plates containing 100 μg/mL ampicillin (Table 2). The inoculated plates were incubated at 37 °C for 18 h (all constant-temperature incubations described in this manuscript were carried out using a Yiheng LRH-250 thermostatic incubator (Shanghai Yiheng Scientific Instrument Co., Ltd., Shanghai, China)) to screen positive clones grown on ampicillin-supplemented LB agar (Table 2) plates.

The positive monoclones were taken from the plates [15], and the plasmid extraction was performed using a plasmid extraction kit (Table 1), according to the manufacturer’s instructions. PCR screening was conducted using 0.5 μL of extracted plasmid as template, with reaction components as listed in Table 6 and TanLpl-F and TanLpl-R primers under the following conditions: initial denaturation at 94 °C for 5 min; followed by 32 cycles of denaturation (94 °C, 30 s), annealing (55 °C, 30 s), and extension (72 °C, 30 s); and then a further extension at 72 °C for 5 min. The amplification products were stored at 4 °C, then separated by agarose gel electrophoresis. The target band was excised from the gel and purified using an agarose gel DNA recovery kit (Table 1), with the process conducted according to the standard protocol.

The recombinant plasmids were verified by DNA sequencing, and only those with the correct sequence were used for the construction of recombinant strains [16,17].

#### 2.3.2. Construction of Plasmid TanLpl-pHT43

The *TanLpl* sequence was amplified in PCR with TanLpl-F and TanLpl-R primers using *Lactiplantibacillus plantarum* ATCC14917T genomic DNA, prepared as above, as template. The amplified product was then electrophoresed on agarose gel, following the manufacturer’s protocol, and the target band was extracted and purified twice, following the manufacturer’s protocol. This was followed by fusing TanLpl-p43-F/TanLpl-p43-R. PCR was performed using the *TanLpl* amplicon as the template, with primers containing the P43 promoter, and the target band was recovered and purified for downstream use. The pHT43 vector backbone was individually amplified from the pHT43 plasmid using primers pHT43-HindIIIF and p43-R, followed by gel electrophoresis and the purification of the target band.

The purified *TanLpl* amplicon and the pHT43 vector backbone PCR products were ligated using a recombinant cloning kit of Easy Geno Assembly Mix (Table 1), according to the manufacturer’s protocol. The reaction mixtures are described in Table 7. The recombinant plasmid was then subjected to competent cell transformation into *E. coli* DH5α competent cells. The preparation of competent cells and the transformation of *B. subtilis* were performed according to standard procedures [15]. These were subsequently transferred onto plates of LB agar supplemented with 100 μg/mL ampicillin (Table 2). The inoculated plates were incubated at 37 °C for 18 h to screen positive clones grown on ampicillin-supplemented LB agar (Table 2) plates.

The positive monoclones were taken from the plates [15], and the plasmid extraction was performed using a plasmid extraction kit (Table 1), according to the manufacturer’s instructions. PCR screening was conducted using 0.5 μL of extracted plasmid as template, with reaction components listed in Table 5 and TanLpl-F and TanLpl-R primers under the following conditions: initial denaturation at 94 °C for 5 min; followed by 32 cycles of denaturation (94 °C, 30 s), annealing (55 °C, 30 s), and extension (72 °C, 30 s); and then a further extension at 72 °C for 5 min. The amplification products were stored at 4 °C, then separated by agarose gel electrophoresis. The target band was excised from the gel and purified using an agarose gel DNA recovery kit (Table 1), with the process conducted according to the standard protocol.

The recombinant plasmids were verified by DNA sequencing, and only those with the correct sequence were used for the construction of recombinant strains [16,17].

### 2.4. Recombinant Strain Construction

#### 2.4.1. Preparation of *Bacillus subtilis* Competent Cells

Streak *Bacillus subtilis* 168(BS 168) and *Bacillus subtilis* WB600(BS WB600) onto LB agar plates (Table 2), and incubate invertedly at 37 °C for 24 h. Take single colonies from the streaked plates [17] and inoculate each into LB liquid medium (Table 2). Shake-culture at 150 rpm/min and 37 °C for 16 h. Transfer the cultures into fresh growth medium (Table 2) at a 1:12 (*v*/*v*) inoculum ratio. The cultures were shake-cultured at 150 rpm and 37 °C until the OD_600_ = 0.9 (all incubations at constant temperature with shaking described in this manuscript were carried out Using a DHZ-DA full-temperature shaking incubator (Taicang Haocheng Experimental Instrument Manufacturing Co., Ltd., Taicang, Suzhou, China)). Chill the culture on ice for 10 min. Then, centrifuge the culture at 5000 rpm for 5 min at 4 °C to harvest the cell pellet. Wash the cell pellet three times with ice-cold electroporation buffer (Table 2, EP buffer) to remove residual medium. Resuspend the cell pellet with 1.2 mL electroporation buffer to prepare competent cells. Aliquot the competent cells and store at −80 °C in a DW-86L728BPST ultra-low temperature freezer (Haier Biomedical Co., Ltd., Qingdao, China).

#### 2.4.2. Electroporation of Plasmid TanLpl-p43NMK

Add 150 μL of BS168 competent cells (Section 2.4.1) as the expression host into a pre-chilled electroporation cuvette, followed by the addition of 5 μL of the previously prepared linearized plasmid TanLpl-p43NMK (Section 2.3.1). After mixing thoroughly, incubate the mixture on ice for 5 min. Apply an electrical pulse at 2.0 kV for 4.2 ms. Immediately after electroporation, add 1 mL of electroporation recovery medium (Table 2) and incubate with shaking at 37 °C and 100 r/min for 3 h. Plate 100 μL of the culture onto LB agar plates containing 20 μg/mL kanamycin (Table 2). The plates were incubated invertedly at 37 °C for 24 h. Positive colonies of *Bacillus subtilis* 168 harboring the recombinant plasmid that could grow on kanamycin-supplemented LB agar plates were screened and designated as BS168 (TanLpl-p43NMK). Extract genomic DNA from positive colonies using a DNA extraction kit (Table 2). The p43NMK sequence was amplified in p43NMK-F and p43NMK-R primers using BS168 (TanAn-p43NMK) genomic DNA, prepared as above, as template. PCR-verified clones containing the correctly integrated genes were used for inoculation [17].

#### 2.4.3. Electroporation of Plasmid TanLpl-pHT43

Separately add 150 μL of BS 168 and BS WB600 competent cells (Section 2.4.1) as the expression hosts into a pre-chilled electroporation cuvette, followed by the addition of 5 μL of the previously prepared linearized plasmid TanLpl-pHT43 (Section 2.3.2). After mixing thoroughly, incubate the mixture on ice for 5 min. Apply an electrical pulse at 2.0 kV for 4.2 ms. Immediately after electroporation, add 1 mL of electroporation recovery medium (Table 2) and incubate with shaking at 37 °C and 100 r/min for 3 h. Plate 100 μL of the culture onto LB agar plates containing 15 μg/mL chloramphenicol (Table 2). The plates were incubated invertedly at 37 °C for 24 h. Separately, positive colonies of *Bacillus subtilis* 168 and *Bacillus subtilis* WB600 harboring the recombinant plasmid that could grow on chloramphenicol-supplemented LB agar plates were screened and designated as BS168(pHT43) and WB600(pHT43). Extract genomic DNA from positive colonies using a DNA extraction kit (Table 2). The pHT43 sequence was amplified in pHT43-F and pHT43-R primers using BS168(pHT43) and WB600(pHT43) genomic DNA, prepared as above, as template. PCR-verified clones containing the correctly integrated genes were used for inoculation [17].

### 2.5. Expression Verification Protocol for Recombinant Strains

Inoculate strains BS168(TanLpl-p43NMK) in LB liquid medium supplemented with 20 μg/mL kanamycin (Table 2) at 37°C with shaking (120 rpm) for 18 h. The samples were centrifuged at 4 °C and 5000 rpm for 5 min, and the supernatant was collected as the crude tannase enzyme. SDS-PAGE analysis was performed.

Separately, incubate strains BS168(pHT43) and WB600(pHT43) in LB liquid medium supplemented with 15 μg/mL chloramphenicol (Table 2) at 37°C with shaking (120 rpm) for 18 h. After the cultivation of BS168(pHT43) and WB600(pHT43), IPTG (Table 2) was added to a final concentration of 1 mM, followed by induction for 6 h. The samples were centrifuged at 4 °C and 5000 rpm for 5 min, and the supernatant was collected as the crude tannase enzyme. SDS-PAGE analysis was performed.

Combine each crude tannase enzyme with 4 × SDS loading buffer (Table 1), boil at 100 °C for 10 min, and immediately chill on ice for 5 min. Centrifuge the samples at 4 °C, 12,000 rpm/min for 10 min to remove debris. The tannase protein expression was assessed by 12% SDS-PAGE gels (Table 1), followed by Coomassie Brilliant Blue staining (Table 1) for visualization [17].

### 2.6. Determination of Growth Characteristics of Recombinant Strains

Inoculate strain BS168(p43NMK) in LB liquid medium supplemented with 20 μg/mL kanamycin (Table 2). Separately inoculate strains BS168(pHT43) and WB600(pHT43) in LB liquid medium supplemented with 15 μg/mL chloramphenicol. Incubate all cultures at 37 °C with shaking at 150 rpm/min. Cultures were sampled every 2 h to measure the OD_600_ value, and then the growth curves were plotted.

### 2.7. Determination of Expression Stability in Recombinant Strains

Recombinant strains were inoculated into LB liquid medium (Table 2) at 37 °C with shaking at 150 rpm/min for 24 h, followed by 10 consecutive serial passages. During each passage, aliquots were collected and serially diluted using a gradient dilution method [18]. The diluted samples were then plated onto LB agar plates (Table 2) and antibiotic-supplemented LB agar plates. Strains carrying the p43NMK plasmid were plated on LB agar containing kanamycin (Table 2). Strains carrying the pHT43 plasmid were plated on LB agar containing chloramphenicol (Table 2). Each dilution gradient was plated in triplicate. After 24 h of inverted incubation at 37 °C, colonies were counted to calculate the plasmid loss rate using the following formula:

Plasmid loss rate (%) = [(Colonies on antibiotic-free plates − Colonies on antibiotic plates)/Colonies on antibiotic-free plates] × 100%.

### 2.8. Enzymatic Characterization of Tannase from Recombinant Strains

#### 2.8.1. Determination of Optimum Temperature for Enzymatic Reaction

Inoculate strain BS168(p43NMK) in LB liquid medium supplemented with 20 μg/mL kanamycin (Table 2) and incubate at 37 °C with shaking at 100 rpm for 24 h for later use. Separately inoculate strains BS168(pHT43) and WB600(pHT43) in LB liquid medium containing 15 μg/mL chloramphenicol (Table 2), followed by incubation under identical shaking conditions (37 °C, 100 rpm) for 18 h. Induce protein expression by adding IPTG to a final concentration of 1 mM for 6 h.

Harvest cultures via centrifugation at 3000 rpm for 10 min and collect supernatants. Incubate supernatants at 20 °C, 25 °C, 30 °C, 35 °C, 40 °C, 45 °C, 50 °C, and 55 °C, maintaining each temperature for 2 h. Measure tannase activity using the Tannase Assay Kit (Micro method; Beijing Solarbio Science & Technology (Beijing, China) Co., Ltd.), strictly following the manufacturer’s protocol. Calculate relative enzyme activity ratios using the highest activity value as 100% baseline. The temperature corresponding to the maximum activity ratio is determined as the optimum temperature for enzymatic reactions.

#### 2.8.2. Determination of Optimum pH for Enzymatic Reactions

Inoculate strain BS168(p43NMK) in LB liquid medium supplemented with 20 μg/mL kanamycin and incubate at 37 °C with shaking at 100 rpm for 24 h. Separately inoculate strains BS168(pHT43) and WB600(pHT43) in LB liquid medium containing 15 μg/mL chloramphenicol, followed by incubation under identical shaking conditions (37 °C, 100 rpm) for 18 h. Induce protein expression by adding IPTG to a final concentration of 1 mM for 6 h. Harvest cultures via centrifugation at 3000 rpm for 10 min and collect supernatants. Adjust the pH of supernatants to 2, 3, 4, 5, 6, 7, 8, and 9, maintaining each pH condition for 2 h. Measure tannase activity using the Tannase Assay Kit (Micro method; Beijing Solarbio Science & Technology Co., Ltd.), strictly following the manufacturer’s protocol. This kit uses propyl gallate (PG) as the standard substance. One unit of tannase activity is defined as the amount of enzyme that degrades 1 nmol of PG per minute in the reaction system per 10^4^ bacterial cells. Calculate relative enzyme activity ratios using the highest activity value as 100% baseline. The pH value corresponding to the maximum activity ratio is identified as the optimum pH for enzymatic reactions [19].

### 2.9. Data Analysis

Data organization was performed using Microsoft Excel 2019 (Microsoft Corporation, Redmond, WA, USA). Data analysis was carried out via SAS 9.4 (SAS Institute Inc., Cary, NC, USA), Origin 2021 (OriginLab Corporation, Northampton, MA, USA) and R 4.2.1 (R Core Team). Three biological replicates and three technical replicates were arranged in this experiment. A one-way analysis of variance was used for data analysis, and different superscript letters represented significant differences (*p* < 0.05).

## 3. Results

### 3.1. Construction of Recombinant Plasmids

**Figure 1:**
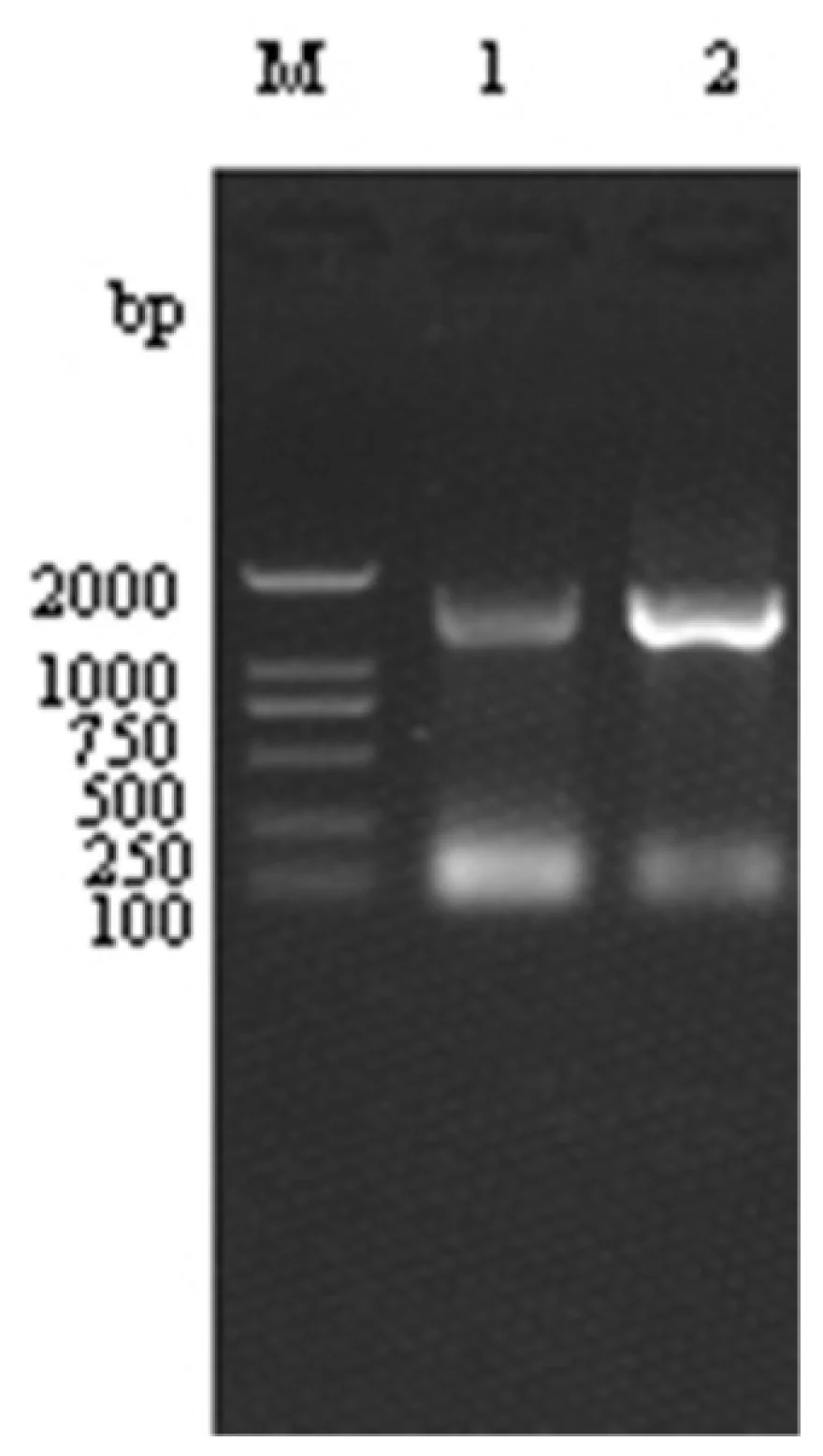
PCR identification of recombinant vector. Figure legend—M: Marker, 1: TanLpl-p43NMK, 2: TanLpl-pHT43.

The PCR verification showed bright, smearing-free characteristic bands, with molecular weights of approximately 1590 bp (Figure 1) for the constructed recombinant plasmids. This demonstrated successful constructions of the recombinant plasmids that were designated as TanLpl-p43NMK and TanLpl-p43NMK.

### 3.2. Construction of Recombinant Strains

**Figure 2:**
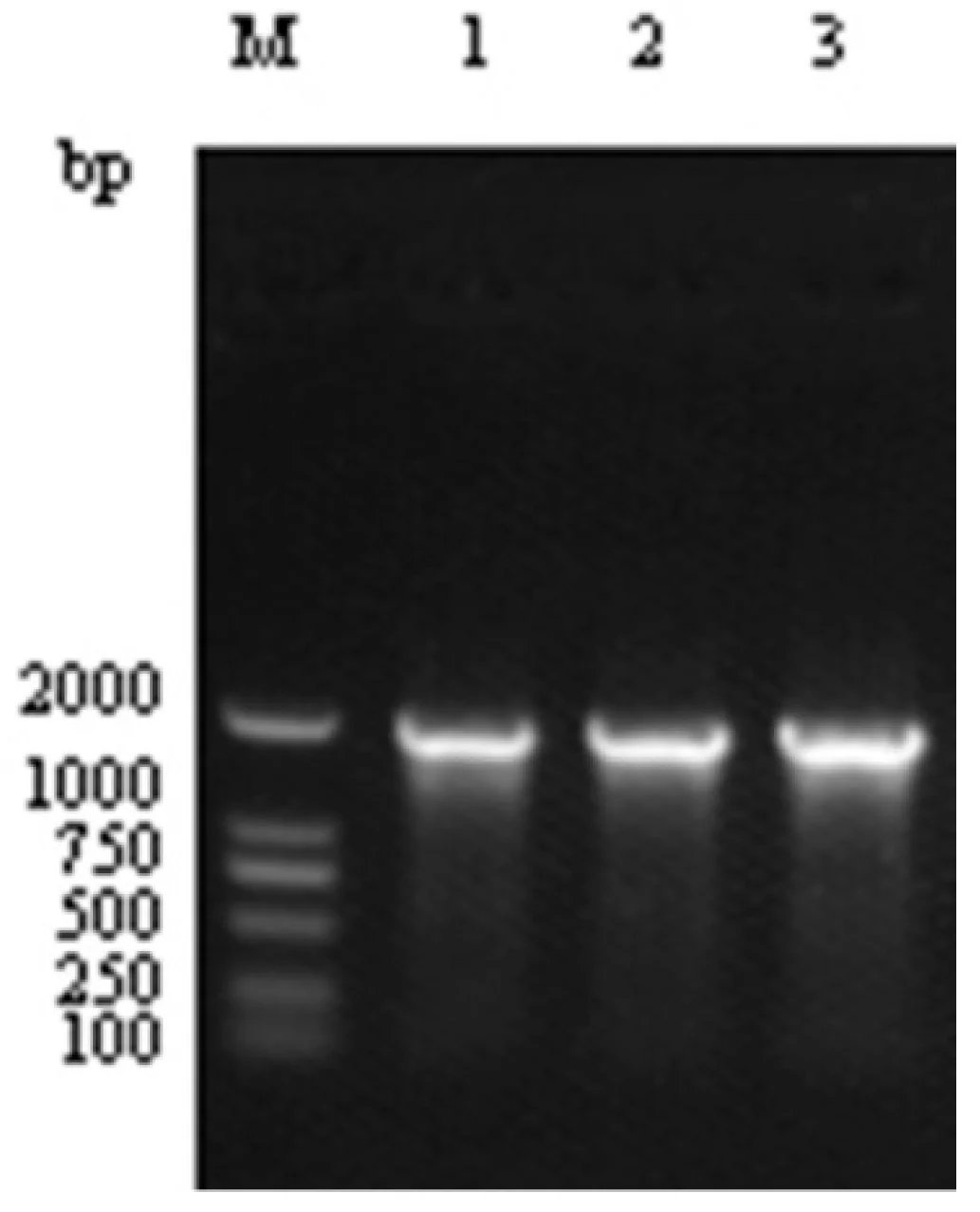
PCR identification of random positive clones. Figure legend—M: Marker, 1: BS168(TanLpl-p43NMK), 2: BS168(pHT43), 3: WB600(pHT43).

PCA amplification yielded bright, smearing-free characteristic bands of BS168 with molecular weights of approximately 1590 bp (Figure 2) for TanLpl-p43NMK and BS168 and WB600 (molecular weight; Figure 2) for TanLpl-pHT43. These results showed a successful transformation of plasmid TanLpl-p43NMK into BS168, designated as BS168(TanLpl-p43NMK). Additionally, the transformations of TanLpl-pHT43 into BS168 and WB600 were designated as BS168(TanLpl-pHT43) and WB600(TanLpl-pHT43).

### 3.3. Expression Profiling of Recombinant Strains

**Figure 3:**
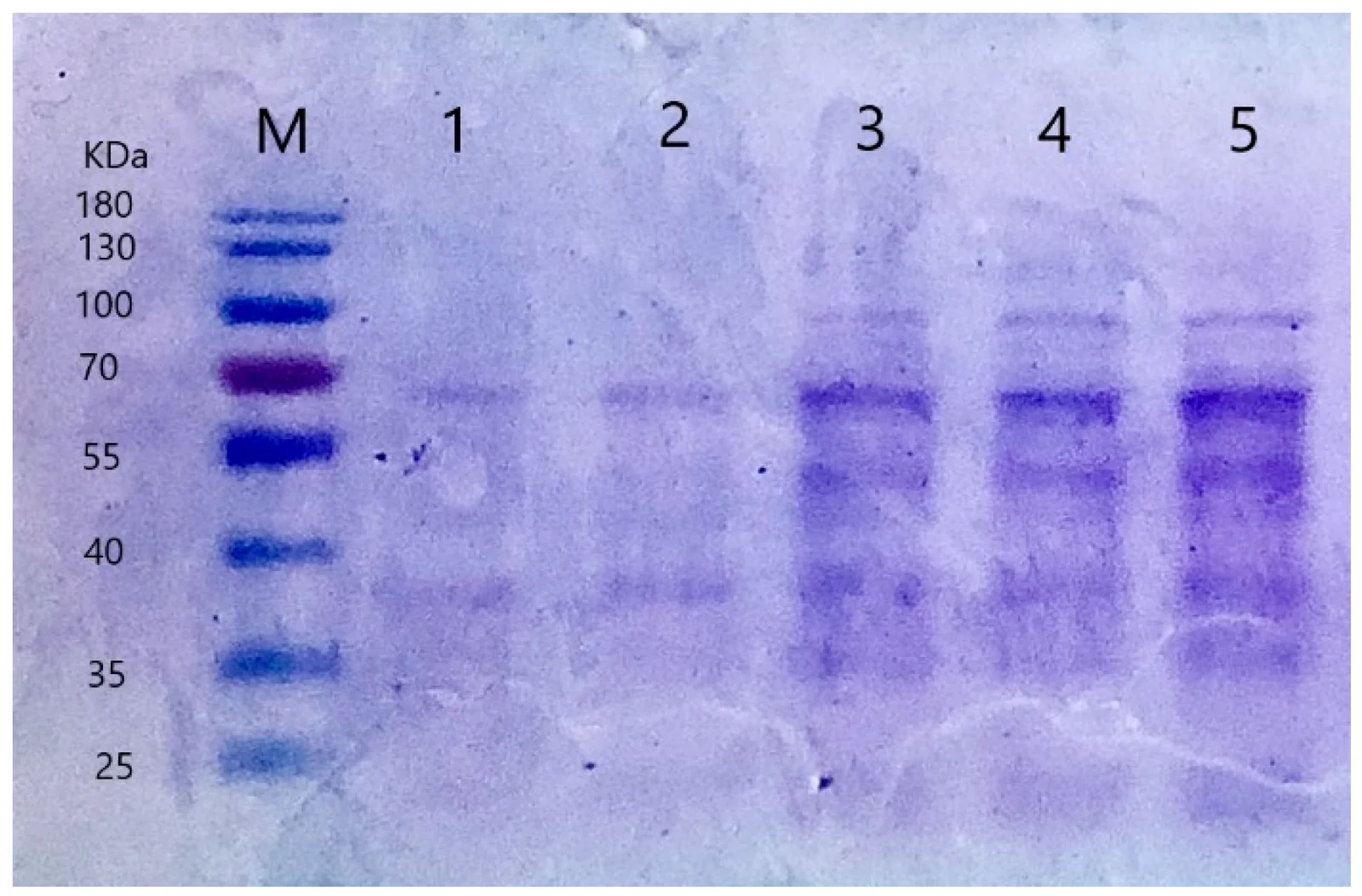
BS168(p43NMK), BS168(pHT43) and WB600 (pHT43) SDS-PAGE electrophoretogram. Figure legend—M: Marker, 1: BS168, 2: WB600, 3: BS168(TanLpl-p43NMK), 4: BS168(pHT43), 5: WB600(pHT43).

BS168(TanLpl-p43NMK), BS168(pHT43), and WB600(pHT43) all exhibited distinct bands at the expected size of approximately 50 kDa (Figure 3). These results indicate the successful expression of the recombinant plasmid TanLpl-p43NMK in BS168 and the successful expression of the recombinant plasmid TanLpl-pHT43 in both BS168 and WB600. Notably, BS168(p43NMK) required no induction, whereas BS168(pHT43) and WB600(pHT43) transformants necessitated induction with 1 mM IPTG for 6 h.

### 3.4. Growth Characteristics of Recombinant Strains

**Figure 4:**
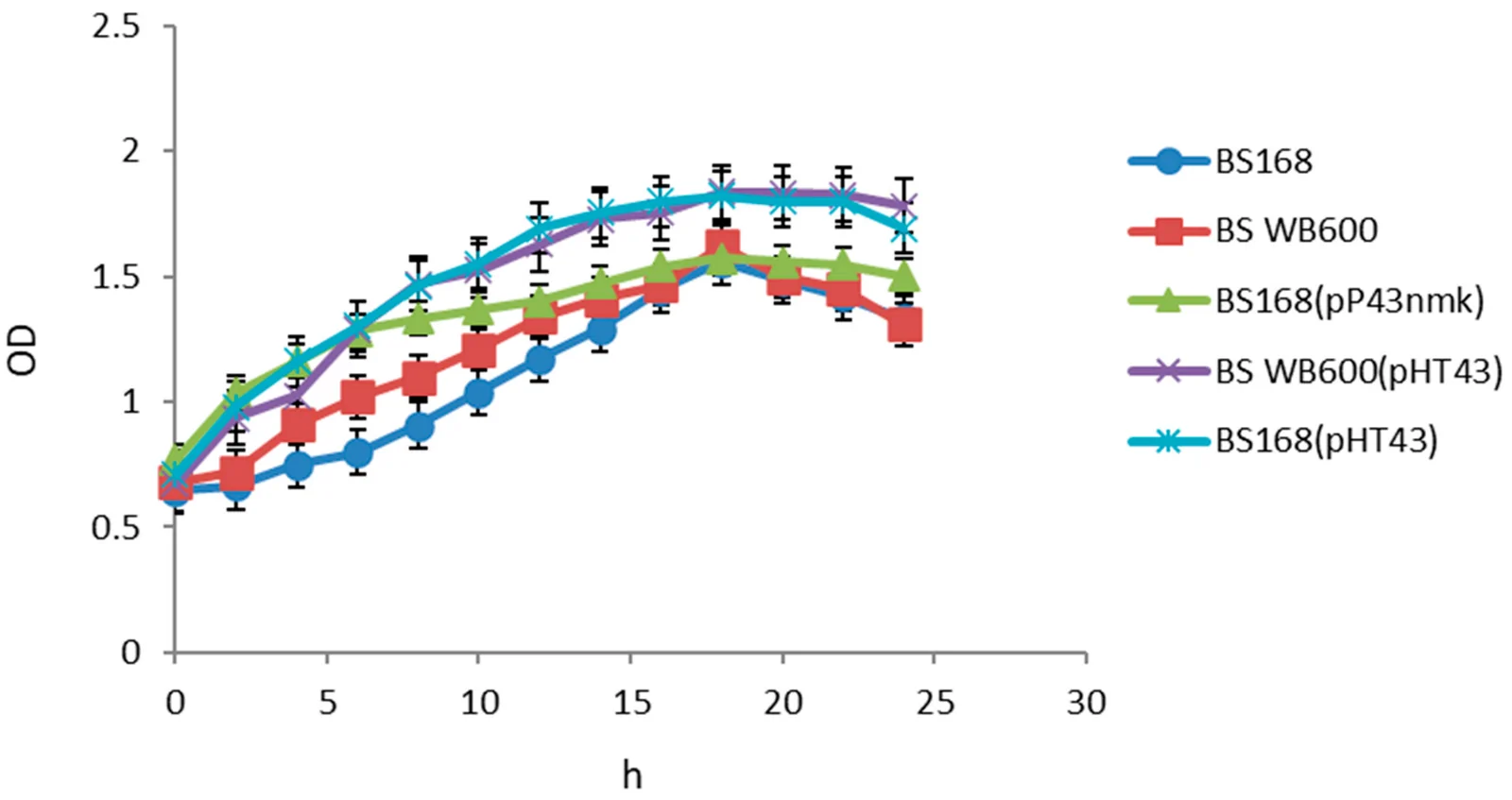
Growth curves of BS168, WB600, BS168 (p43NMK), BS168(pHT43), WB600 (pHT43).

The five recombinant strains (BS168, WB600, BS168(p43NMK), BS168(pHT43) and WB600(pHT43)) exhibited similar growth kinetics, entering the stationary phase after approximately 18 h (Figure 4), and had similar growth rates to that of the parental strain.

### 3.5. Expression Stability of Recombinant Strains

There was a significant difference in the plasmid loss rates among the three recombinant strains (*p* < 0.001; Table 8). The plasmid loss rates of WB600 (pHT43) in the 1st and 2nd generations was higher (*p*-value *p* < 0.001; Table 8) than those of BS168(pHT43) and BS168(p43NMK). The plasmid loss rates of the three recombinant strains increased as the passage number increased. At the 10th passage, the plasmid loss rates of BS168(pHT43) and WB600 (pHT43) were significantly lower (*p*-value) than that of BS168(p43NMK), with BS168(p43NMK) being approximately twice those of BS168(pHT43) and WB600 (pHT43). These results indicate that the recombinant strains BS168(pHT43) and WB600 (pHT43) can stably inherit the carried target gene.

### 3.6. Enzymatic Properties of Tannase from Recombinant Strains

**Figure 5:**
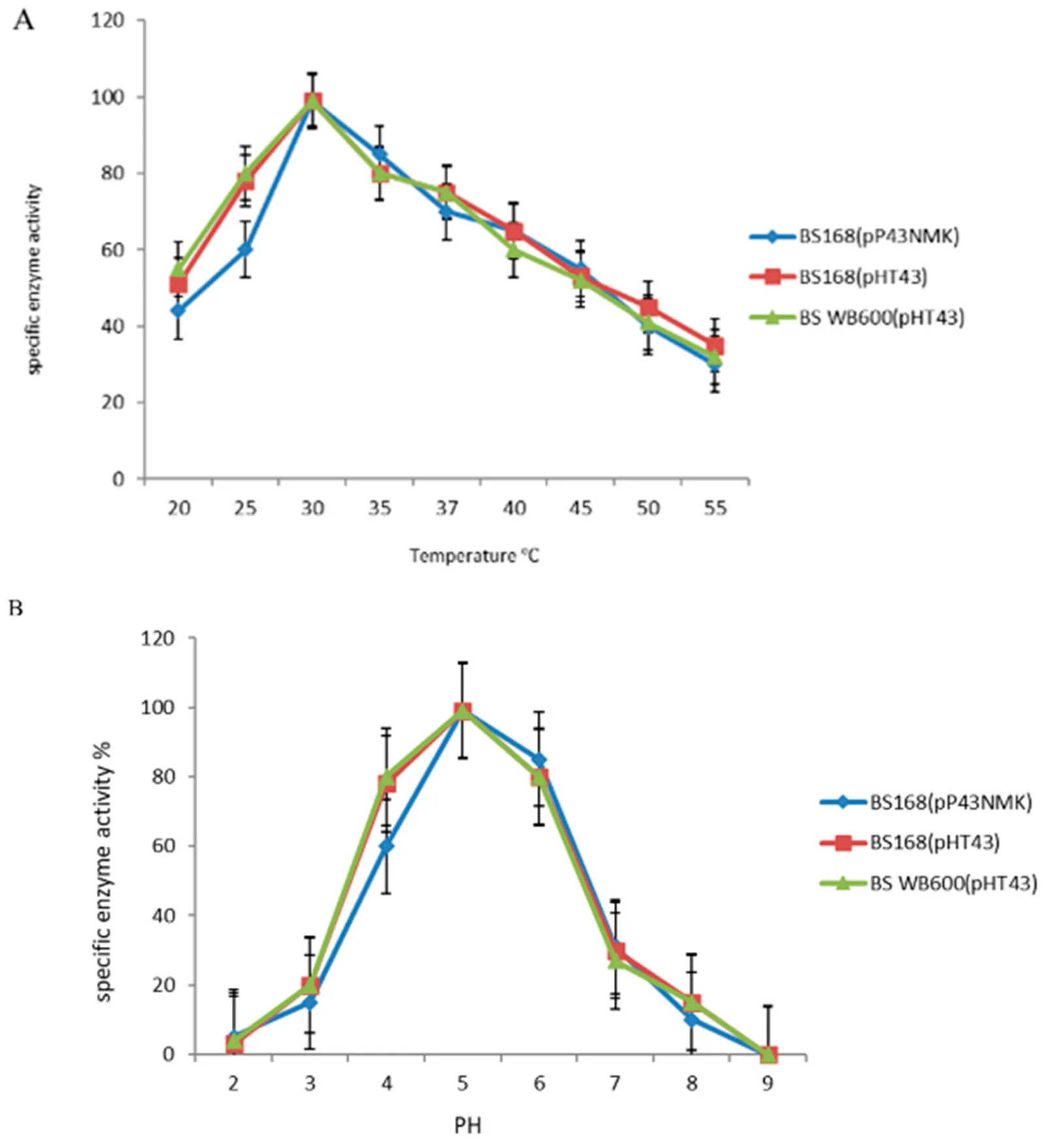
Effect of temperature (**A**) and pH (**B**) on the tannase activities of BS168(p43NMK), BS168(pHT43) and WB600(pHT43).

The effect of temperature on tannase activity produced by the three recombinant strains showed similar curves, and the optimal enzymatic reaction temperature was uniformly 30 °C (Figure 5A). The tannase relative activity increased as the temperature increased from 20 °C to 30 °C, reaching a maximal value of 100% at temperature = 30 °C, and thereafter gradually decreased as the temperature increased to 55 °C.

The optimal pH for the enzymatic reaction of tannase produced by the three recombinant strains was 5.0 (Figure 5B). The tannase relative activity increased as the pH increased from 2 to 5, reaching a maximal value of 100% at pH = 5, and thereafter gradually decreased as the pH increased to 9.

### 3.7. Tannase Activity of Recombinant Strains Under Optimal Conditions

Under the optimal conditions (pH 5.0, 30 °C), the tannase production activities of BS168(p43NMK), BS168(pHT43) and WB600(pHT43) were 68.81, 397.36 and 461.12 U/mL, respectively (Table 9).

## 4. Discussion

Tannase-producing microorganisms are widely distributed in nature, with fungi and bacteria being the primary natural sources [1,2]. Although fungi possess relatively strong tannase-producing capabilities, they are not conducive to the preservation of fermented feed and exhibit poor aerobic stability [20], which imposes certain limitations on their application in industrial production. On the other hand, the low enzyme yield of bacteria fails to meet the demands of fermented feed production [21]. Therefore, the efficient heterologous expression of tannase represents a viable and effective strategy to address this bottleneck.

The *Bacillus subtilis* expression system consists of Generally Recognized as Safe (GRAS) microorganisms and is classified as a food-safe strain [13]. *B. subtilis* is non-pathogenic, with a single-layer outer membrane that grows rapidly with low nutritional requirements and that can directly secrete many extracellular proteins [22]. It possesses a well-defined genetic background and complete genome information, with a wealth of plasmid expression systems, genome-editing tools, and gene expression regulatory modules available [23]. *B. subtilis* was the first Bacillus species found to exhibit natural competence [24]. The *B. subtilis* expression system has been used for the efficient secretory expression of many heterologous proteins, with B. subtilis 168 and its derivatives being the most commonly used strains [25].

However, the *B. subtilis* expression system can secrete large amounts of extracellular proteases in the stationary phase, which can degrade the target protein [22]. *B. subtilis* WB600 carries deletions of six extracellular protease genes (nprE, nprB, aprE, epr, mpr, bpr), and its extracellular proteolytic activity is less than 0.32% of that of wild-type *B. subtilis* [26]. Therefore, in this study, *B. subtilis* 168 and *B. subtilis* WB600 were used as hosts for the efficient heterologous expression of the tannase gene: TanLpl from Lactiplantibacillus plantarum ATCC 14917ᵀ.

This study showed that TanLpl was successfully expressed with detectable extracellular enzyme activity. Under the optimal conditions (30 °C, pH 5.0), the tannase activity produced by *B. subtilis* WB600(pHT43) reached 461.12 U/mL, which was superior to that of *B. subtilis* 168(p43NMK) and *B. subtilis* 168(pHT43) because, compared with p43NMK, pHT43 possesses stronger IPTG-inducible Pgrac promoter and high-efficiency amyQ signal peptide, which can significantly enhance gene transcription and extracellular protein secretion. Meanwhile, pHT43 exhibits a higher plasmid copy number and better genetic stability. In addition, *Bacillus subtilis* WB600 is deficient in multiple extracellular proteases, which effectively avoids the degradation of secreted tannase and further improves the extracellular expression level of target proteins [27]. This activity was higher than the activity of the tannase gene Gt-Tan sequence recombinantly expressed in Escherichia coli BL21 [6], and also higher than that of the native tannase-producing strain *A. niger* N5-5 [28]. The optimal fermentation temperature commonly employed in fermented feed production is generally in the range of 30~35 °C [29], which is highly consistent with the optimal temperature of 30 °C for tannase activity determined in this study. Meanwhile, the pH of fermented feed usually decreases to approximately 4.5~5.5 during fermentation, which is also highly compatible with the optimal pH 5.0 of the recombinant tannase. These results suggest that the environmental conditions of conventional fermented feed can satisfy the requirements for both the growth of *B. subtilis* WB600 and the catalytic activity of TanLpl. Therefore, the recombinant strain constructed in this study exhibits good application potential in the production of tannin-rich fermented feed, as it can achieve efficient enzyme expression and exert its catalytic function synchronously with the feed fermentation process.

*B. subtilis* WB600 has also been successfully used for recombinant expressions of other proteins, for example, pullulanase gene, with enzyme yield being 5.5-fold higher than that in recombinant *E. coli* [30]; phosphorylase gene [31]; and keratinase gene, with the extracellular enzyme activity being 15.2-fold higher than that of the parental strain [32]. These improvements may be attributed to the deletion of extracellular proteases in *B. subtilis* WB600, allowing the stable expression and secretion of heterologous extracellular proteins. All together, these results demonstrate that *B. subtilis* WB600 is more suitable as an expression system for the expression of heterologous extracellular proteins [33,34].

pHT43 is an *E. coli*–*Bacillus subtilis* shuttle vector constructed by ligating the endogenous θ-replicating plasmid replicon of *B. subtilis* with an *E. coli* cloning vector, which has exhibited good stability in *B. subtilis* [35]. In this study, when *B. subtilis* 168 was used as the expression host, the tannase activity of the recombinant strain harboring pHT43 as the expression vector was 5.7-fold higher than that of the recombinant strain with p43NMK as the expression vector. These results suggest that pHT43 constructed in this study possesses superior expression efficiency and stronger secretion capacity for tannase compared with p43NMK, making it a more suitable shuttle vector for the heterologous expression and extracellular production of tannase in *Bacillus subtilis.* Although the strain has certain theoretical application prospects in fermented feed production, large-scale solid-state fermentation simulation experiments and actual production verification are still lacking. And follow-up simulated feed fermentation tests will be carried out to further confirm its practical application effect.

The results of the recombinant strain expression stability showed that, starting from the 3rd passage, the plasmid loss rate of *B. subtilis* 168(p43NMK) was higher than those of *B. subtilis* 168(pHT43) and *B. subtilis* WB600(pHT43), and reached 98.72% at the 10th passage, which was twice those of the other two recombinant strains. These findings indicate that plasmid pHT43 is superior to plasmid p43NMK in both expression stability and expression capacity.

In summary, the high-tannase-producing recombinant strain *B. subtilis* WB600(pHT43), constructed using pHT43 as the expression vector and *B. subtilis* WB600 as the expression host, exhibited superior enzyme production capacity and stability compared to the other two recombinant strains. The optimal conditions for the extracellular tannase secreted by the recombinant strain were similar to those of fermented feed production, indicating that this recombinant strain can be used as a microbial agent for *Caragana korshinskii* fermentation.

## 5. Conclusions

(1) The TanLpl gene sequence from *Lactiplantibacillus plantarum* ATCC 14917ᵀ was inserted into the *Bacillus subtilis* expression system, yielding three stable tannase-producing recombinant strains: *B. subtilis* 168(p43NMK), *B. subtilis* 168(pHT43), and *B. subtilis* WB600(pHT43).
(2) Under optimal conditions (pH 5.0, 30 °C), the tannase activities of the three recombinant strains were 68.81, 397.36 and 461.12 U/mL, respectively. The recombinant strain *B. subtilis* WB600(pHT43) was superior to the other two strains in terms of expression stability and enzyme production capacity.

## 6. Future Prospects

(1) In future studies, more kinds of metal ions and chemical additives will be tested to further explore the stability and catalytic adaptability of recombinant tannase.
(2) Optimized fermentation conditions will be explored to improve the extracellular secretion efficiency and industrial production level of this enzyme.
(3) This tannase will be applied in feed processing and agricultural by-product utilization to realize practical popularization and application.

## Figures and Tables

**Table 1:** Plasmids and strains.

Name	Source
*E. coli*/*B. subtilis* shuttle vector p43NMK	Hangzhou Baosai Biotechnology (Hangzhou, China) Co., Ltd.
*E. coli B. subtilis* shuttle vector pHT43	Hangzhou Baosai Biotechnology (Hangzhou) Co., Ltd.
*E.coli* DH5α competent cells	Hangzhou Baosai Biotechnology (Hangzhou) Co., Ltd.
*Bacillus subtilis* 168	In-house strain
*Bacillus subtilis* WB600	In-house strain
*Lactiplantibacillus plantarum* ATCC14917T	American Type Culture Collection (ATCC)
T4 DNA ligase	Takara Biomedical Technology (Beijing, China) Co., Ltd.
Isopropyl-β-D-thiogalactopyranoside (IPTG)	Takara Biomedical Technology (Beijing) Co., Ltd.
DL2000 DNA marker	Hangzhou Baosai Biotechnology (Hangzhou) Co., Ltd.
2 × Superpfu PCR mix	Hangzhou Baosai Biotechnology (Hangzhou) Co., Ltd.
2 × Taq PCR mix	Hangzhou Baosai Biotechnology (Hangzhou) Co., Ltd.
2 × pfu PCR mix	Hangzhou Baosai Biotechnology (Hangzhou) Co., Ltd.
5 × Seamless Cloning mix	Hangzhou Baosai Biotechnology (Hangzhou) Co., Ltd.
λDNA HindIII digest DNA marker	Thermo Fisher Scientific, Waltham, WA, USA
4 × SDS loading buffer	TIANGEN Biotech (Beijing, China) Co., Ltd.
SDS-PAGE gels	TIANGEN Biotech (Beijing) Co., Ltd.
Coomassie Brilliant Blue	TIANGEN Biotech (Beijing) Co., Ltd.
Agarose gel DNA recovery kit	TIANGEN Biotech (Beijing) Co., Ltd.
Plasmid extraction kit	TIANGEN Biotech (Beijing) Co., Ltd.
Easy Geno Assembly Mix	TIANGEN Biotech (Beijing) Co., Ltd.

**Table 2:** Culture media and electroporation buffer.

Medium	Sources or Compositions (g/L)
LB liquid medium	Guangdong Huankai Microbial Science & Technology (Guangzhou, China) Co., Ltd.
LB agar medium	Guangdong Huankai Microbial Science & Technology (Guangdong) Co., Ltd.
Potato dextrose broth (PDB)	Guangdong Huankai Microbial Science & Technology (Guangdong) Co., Ltd.
Potato dextrose agar (PDA)	Guangdong Huankai Microbial Science & Technology (Guangdong) Co., Ltd.
LB liquid medium supplemented with 20 μg/mL kanamycin	Guangdong Huankai Microbial Science & Technology (Guangdong) Co., Ltd.
LB liquid medium supplemented with 15 μg/mL chloramphenicol	Guangdong Huankai Microbial Science & Technology (Guangdong) Co., Ltd.
LB agar medium supplemented with 20 μg/mL kanamycin	Guangdong Huankai Microbial Science & Technology (Guangdong) Co., Ltd.
LB agar medium supplemented with 15 μg/mL chloramphenicol	Guangdong Huankai Microbial Science & Technology (Guangdong) Co., Ltd.
LB liquid medium supplemented with 100 μg/mL ampicillin	Guangdong Huankai Microbial Science & Technology (Guangdong) Co., Ltd.
LB agar medium supplemented with 100 μg/mL ampicillin	Guangdong Huankai Microbial Science & Technology (Guangdong) Co., Ltd.
Growth medium	tryptone 10, yeast extract 5, NaCl 10, sorbitol 0.5 mol
Electroporation buffer	sorbitol 0.5 mol, mannitol 0.5 mol, glycerol 10%.
Electroporation recovery medium	tryptone 10, yeast extract 5, NaCl 10, sorbitol 0.5 mol, mannitol 0.38 mol

**Table 3:** PCR primer information.

Primer Name	Primer Sequence (5′→3′)
TanLpl-F	ATGAGTAACCGATTGATTTTTGA
TanLpl-R	TCATTGGCACAAGCCATCAATCC
TanLpl-p43-F	GGTACCAAGAGAGGAATGTACACATGAGTAACCGATTGATTTTTGA
TanLpl-p43-R	GATTACGCCAAGCTTTTATCATTGGCACAAGCCATCAATCC
p43NMK-hindIIIF	AAGCTTGGCGTAATCATGGTC
pHT43-hindIIIF	CCAAGCTTAAAGGAGGACACGCATGAGTTC
p43-R	TGTACATTCCTCTCTTGGTACCGCTATCACTTTATATT
p43NMK-F	GTATGTTTTCGCTTGAACTTTTA
p43NMK-R	AGCTGGCACGACAGGTTTCCCGA
pHT43-F	TTGCGGTTTCAGCGTATTG
pHT43-R	GGCTCAGCGCCTGTTCTT

**Table 4:** PCR amplification system.

Composition Components in Molecular Biology Protocols	Volume Specifications in Molecular Biology Protocols
2 × pfu PCR mix	25 μL
Upstream primer	2 μL
Reverse primer	2 μL
DNA template	2 μL
ddH_2_O	19 μL
Total reaction volume	50 μL

**Table 5:** Seamless clone link reaction system.

Composition Components in Molecular Biology Protocols	Volume Specifications in Molecular Biology Protocols
5 × seamless cloning mix	2 μL
Gel-purified *TanLpl* amplicon fragment	6 μL
p43NMK backbone fragment	2 μL
Total reaction volume	10 μL

**Table 6:** PCR amplification system.

Composition Components in Molecular Biology Protocols	Volume Specifications in Molecular Biology Protocols
2 × Taq PCR mix	10 μL
Upstream primer	0.5 μL
Reverse primer	0.5 μL
Monoclonal bacterial culture	0.5 μL
ddH_2_O	8.5 μL
Total reaction volume	20 μL

**Table 7:** Seamless clone link reaction system.

Composition Components in Molecular Biology Protocols	Volume Specifications in Molecular Biology Protocols
5 × seamless cloning mix	2 μL
Gel-purified TanLpl amplicon fragment	6 μL
pHT43 backbone fragment	2 μL
Total reaction volume	10 μL

**Table 8:** Plasmid loss rates of recombinant bacteria (%).

Generations	BS168(p43NMK)	BS168(pHT43)	WB600(pHT43)	*p*-Value
1	2.52 ± 0.449 ^c^	6.55 ± 0.310 ^b^	8.69 ± 0.958 ^a^	<0.001
2	5.33 ± 0.109 ^c^	6.57 ± 2.710 ^b^	8.82 ± 1.012 ^a^	<0.001
3	8.05 ± 4.942 ^a^	6.77 ± 0.882 ^b^	7.69 ± 2.027 ^c^	<0.001
4	12.06 ± 0.251 ^a^	11.92 ± 2.111 ^a^	9.31 ± 3.863 ^b^	<0.001
5	24.05 ± 3.163 ^a^	13.86 ± 3.093 ^b^	9.92 ± 0.009 ^c^	<0.001
6	28.03 ± 2.133 ^a^	16.53 ± 0.049 ^b^	14.72 ± 0.161 ^c^	<0.001
7	31.44 ± 3.098 ^a^	18.85 ± 0.629 ^c^	19.14 ± 0.489 ^b^	<0.001
8	55.37 ± 2.109 ^a^	25.75 ± 3.768 ^c^	27.27 ± 3.843 ^b^	<0.001
9	93.07 ± 1.916 ^a^	37.84 ± 1.308 ^b^	30.81 ± 2.189 ^c^	<0.001
10	98.72 ± 1.443 ^a^	44.88 ± 3.117 ^b^	42.11 ± 2.189 ^c^	<0.001

Note: Values followed by different lowercase letters in the same column differ significantly (at *p* < 0.05), while the same letters indicate no significant difference.

**Table 9:** Enzyme activities of recombinant bacteria under optimal conditions.

Strain Designation	Tannase Activity (U/mL)
BS168(p43NMK)	68.81 ± 3.16 ^c^
BS168(pHT43)	397.36 ± 29.33 ^b^
WB600(pHT43)	461.12 ± 10.01 ^a^
*p*-value	<0.001

Note: Values followed by different lowercase letters in the same column differ significantly (at *p* < 0.05), while the same letters indicate no significant difference.

## Data Availability

The data presented in this study are available on request from the corresponding author.
