# Correction: Gao et al. Construction and Evaluation of High-Efficiency Tannase-Producing Strains. *Microorganisms* 2026, *14*, 1233

**DOI:** 10.3390/microorganisms14081672

**Published:** 2026-07-30

**Authors:** Yuan Gao, Chenguang Hu, Wurilege Wei, Delhei Urjid, Yuchao Hu, Xiaojuan Zhao, Yang Liu, Guoqing Guo, Surigalatu Wang, Feng Tian, Jianyong Liang, Jiuyue Li, Hai Jin, Shuyuan Xue

**Affiliations:** 1 Inner Mongolia Academy of Agricultural & Animal Husbandry Sciences, Hohhot 010031, China; may5june6@yeah.net (Y.G.); baron_hcg@sina.com (C.H.); weiwurilege@163.com (W.W.); delhi98@163.com (D.U.); yuchaohu1994@163.com (Y.H.); m15648192392@163.com (X.Z.); 15661271930@163.com (Y.L.); guoqingguo2024@163.com (G.G.); surigalatu2025@163.com (S.W.); tianfeng1309802@126.com (F.T.); liangjy0403@163.com (J.L.); lijy009@163.com (J.L.); jinhaicnm@vip.sina.com (H.J.); 2 Inner Mongolia Agricultural University, Hohhot 010018, China

In the published publication [1], there was an error regarding the affiliation for Yuan Gao. In addition to affiliation **1**, the updated affiliation should include Inner Mongolia Agricultural University. The authors state that the scientific conclusions are unaffected. This correction was approved by the Academic Editor. The original publication has also been updated.
